# Pelagic responses to oceanic anoxia during the Carnian Pluvial Episode (Late Triassic) in Panthalassa Ocean

**DOI:** 10.1038/s41598-023-43525-9

**Published:** 2023-09-28

**Authors:** Yuki Tomimatsu, Tatsuo Nozaki, Tetsuji Onoue, Hironao Matsumoto, Honami Sato, Yutaro Takaya, Jun-Ichi Kimura, Qing Chang, Manuel Rigo

**Affiliations:** 1 Department of Earth and Planetary Sciences, Kyushu University, Fukuoka, 819-0395 Japan; 2 Submarine Resources Research Center, Research Institute for Marine Resources Utilization, Japan Agency for Marine-Earth Science and Technology (JAMSTEC), Kanagawa, 237-0061 Japan; 3 Frontier Research Center for Energy and Resources, The University of Tokyo, Tokyo, 113-8656 Japan; 4 Department of Planetology, Kobe University, Hyogo, 657-8501 Japan; 5 Department of Systems Innovation, The University of Tokyo, Tokyo, 113-8656 Japan; 6 Faculty of Science and Engineering, Waseda University, Tokyo, 169-8555 Japan; 7 Volcanoes and Earth’s Interior Research Center, Research Institute for Marine Geodynamics, Japan Agency for Marine-Earth Science and Technology (JAMSTEC), Kanagawa, 237-0061 Japan; 8 Department of Geosciences, University of Padova, 35131 Padova, Italy; 9 Institute of Geosciences and Earth Resource (IGG–CNR), 35131 Padova, Italy

**Keywords:** Geochemistry, Geochemistry, Palaeoceanography

## Abstract

The Carnian Pluvial Episode (CPE) was a short interval of extreme rainfall in the Late Triassic that caused significant changes in marine ecosystems. Global warming induced by Wrangellia volcanism is thought to have resulted in oceanic anoxia during the CPE, but the global extent, duration, and severity of anoxia, and its effects on major marine taxa, remain unclear. To address this, we examined an equatorial record of conditions in the Panthalassa Ocean during the CPE, focusing on marine Os isotope data, redox conditions, and conodont and radiolarian biostratigraphy. The results show that Wrangellia volcanism peaked in the latest Julian (early Carnian), coinciding with development of reducing conditions in the deep-sea Panthalassa. A strong conodont turnover occurred during the period of oceanic anoxia, whereas radiolarians were less affected and their diversity increased after the recovery from anoxia. The increased radiolarian diversity during the early Tuvalian (late Carnian) can be attributed to chemical weathering and enhanced nutrient fluxes associated with global warming and the more humid climate of Pangea.

## Introduction

During the 50 Myr of the Triassic, the eastern and central regions of Pangea (40°N to 40°S) and the western Tethys received little precipitation and have been classified as having tropical seasonally dry (but wet summer) and subtropical arid desert climates^1, 2^. However, an abrupt change to a dramatically wet climate occurred during the mid-Carnian (late Julian to early Tuvalian; 234–232 Ma) in the Late Triassic. This enigmatic global event was characterized by a geologically short-lived period of increased precipitation, termed the Carnian Pluvial Episode (CPE)^3^. The CPE was characterized by a significant input of siliciclastic material into the ocean that interrupted the growth of carbonate platforms in the Tethys^4, 5^, increased seawater temperatures^6–10^, and resulted in oxygen-depleted conditions in marginal basins^6, 10, 11^. A climatic signal can also be inferred from the pelagic deep-sea chert successions in Japan. The recorded sudden change in mineral composition (lack of chlorite and appearance of smectite) at the JTB occurs due to the increasing humidity in the continental area^12^. In addition, the pelagic successions have described significant mid-Carnian lithological changes during the CPE from bedded cherts to manganese deposits (Supplementary information). It has been suggested that these manganese deposits may have been formed with the changes in marine redox conditions in the CPE^13^.

**Figure 1:**
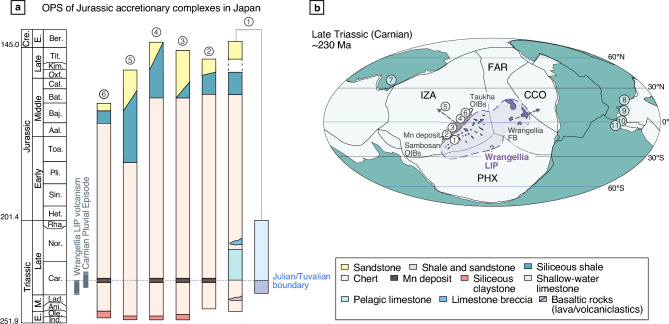
Paleoceanographic setting in the Panthalassa during the CPE. (**a**) Oceanic plate stratigraphy (OPS) of the Panthalassa Ocean reconstructed from Jurassic accretionary complexes in Japan. 1. Sambosan Belt; 2. Chichibu Belt (Takahira section); 3. Tamba Belt (Tamaiwa section); 4. Mino Belt (Kanzaki section); 5. Mino Belt (Sakahogi section); 6. North Kitakami Belt (Otaniyama section). (**b**) Paleogeographic maps showing the hypothetical location of the Wrangellia Large Igneous Province (LIP) during the Late Triassic (Carnian), modified after Tomimatsu et al.^16^. The main marine sections mentioned in the text are also shown. 7. South China Block; 8. Northern Calcareous Alps; 9. Dolomites, Southern Alps; 10. Sicily; 11. Tunisia. Abbreviations: IZA = Izanagi Plate, FAR = Farallon Plate, CCO = Cache Creek Ocean, PHX = Phoenix Plate. The map is created using ACD Systems Canvas Draw software (Version 6.0) (https://www.poladigital.co.jp/canvas/index.html).

These extreme events are thought to have been triggered by the eruption of the Wrangellia flood basalts in the Panthalassa Ocean^14, 15^, as well as the Carnian Panthalassic oceanic island basalt (OIB) volcanism recorded in the Jurassic accretionary complexes of East Asia (i.e., the Sambosan Belt in Japan and the Taukha Belt in the Far East Russia)^16^. The age of these Panthalassic basalts is constrained to the mid-Ladinian to lower Tuvalian based on biostratigraphic data from sedimentary rocks immediately underlying and overlying the basalts^17, 18^. Previous studies of Os and Hg isotopes have also confirmed that this volcanism in the Panthalassa occurred mainly during the late Julian^16, 19, 20^. The coeval emplacement of flood and OIBs in the Panthalassa Ocean suggests the existence of a Carnian large igneous province (LIP), which has been termed the Wrangellia LIP (Fig. 1)^16, 21^. In addition to the Wrangellia LIP, the remnants of the Carnian oceanic basalts of an intraplate origin, have been recognized from Greece to Turkey, Iran(?), and Oman^22^. Their oceanic basalts erupted simultaneously in the Neo-Tethys, which may have formed a large Pan-Arabic LIP in the Carnian^23^. Several contemporaneous Tethyan volcanic events could have contributed to the observed environmental changes in the Carnian triggered by the Wrangelia LIP volcanism.

During the CPE, there were major turnovers of diverse marine taxa in the Tethys Ocean, including crinoids, echinoids, bivalves, bryozoans, ammonoids, radiolarians, gastropods, foraminifers, sponges, brachiopods, corals, ostracods, and conodonts^3, 24, 25^. In particular, the extinction of pelagic fauna, such as ammonoids and conodonts, occurred from the uppermost Julian to the Julian–Tuvalian boundary (JTB), coinciding with the peak in Wrangellia LIP volcanism^26, 27^. In Tethyan regions, possible causal links have been proposed between the extinction events and oceanic anoxia related to global warming caused by Wrangellia LIP volcanism^27, 28^. However, the detailed redox changes in the pelagic environment across the JTB remain unclear, and the factors that led to the pelagic crisis are uncertain.

To better understand the global extent, duration, and severity of oceanic anoxia, and its effects on major pelagic taxa during the CPE, we investigated stratigraphic variations in marine Os isotope compositions (^187^Os/^188^Os), major and trace element compositions of paleo-ocean redox conditions, and the conodont and radiolarian biostratigraphy in Panthalassic pelagic sequences in Japan. Given that marine ^187^Os/^188^Os ratios are mainly controlled by relative balances of continental riverine Os flux (^187^Os/^188^Os ~ 1.4) and the hydrothermal and extraterrestrial Os fluxes (^187^Os/^188^Os ~ 0.12–0.13) into the global ocean, the ^187^Os/^188^Os ratios of pelagic sediments are a robust geochemical indicator of significant volcanism and hydrothermal (unradiogenic Os) inputs into the ocean over geological time^29–35^. Using these data, we show that widespread deep-water anoxic conditions existed in the Panthalassa during the CPE, coinciding with the peak of Wrangellia LIP volcanism. We discuss the effects of these events on the pelagic ecosystem.

## Geological setting

The studied Carnian chert sections are located in the Chichibu, Tamba, Mino, and North Kitakami belts in Japan, which are Jurassic subduction-related accretionary complexes (Supplementary Fig. S1). These complexes consist mainly of thrust sheets of sedimentary sequences containing Triassic to Middle Jurassic bedded chert, and overlying Middle Jurassic to lowermost Cretaceous sandstone and mudstone^36^. The chert sequence is thought to have accumulated in a pelagic, deep-sea setting below the carbonate compensation depth, and records the sedimentary history on an oceanic plate in the Panthalassa Ocean prior to its accretion at the trench (Fig. 1)^37, 38^. Paleomagnetic studies suggest that the Middle and Upper Triassic chert sequences accumulated in an open-ocean setting within the low-latitudinal zone of the mid-Panthalassa^39–41^.

We collected samples from four Carnian chert successions in the Jurassic accretionary complexes: (1) the Takahira section in the Chichibu Belt of western Kyushu; (2) the Tamaiwa section in the Tamba Belt of central Japan; (3) the Kanzaki section in the Mino Belt of central Japan; and (4) the Otaniyama section in the North Kitakami Belt of northwestern Japan (Supplementary Fig. S1). In all the studied sections, the manganese deposits are intercalated within the pelagic chert sequences in the Jurassic accretionary complexes (Supplementary Fig. S2). These widespread near-synchronous depositions of manganese ores in Panthalassa Ocean have been observed during the CPE (upper Julian to lower Tuvalian), based on the radiolarian-conodont biostratigraphy (Fig. 1; Supplementary Fig. S2). Detailed lithostratigraphic and biostratigraphic studies of these sections have been reported by Tomimatsu et al.^13^ (Supplementary Fig. S2). The geological setting of each accretionary complex and brief details of the studied sections are presented in the Supplementary Information. To validate our interpretation of the geochemical records, these sections were also compared with the Sakahogi section in the Mino Belt of central Japan, which was referred to as Section N–O in a previous study^16^.

## Materials and methods

**Figure 2:**
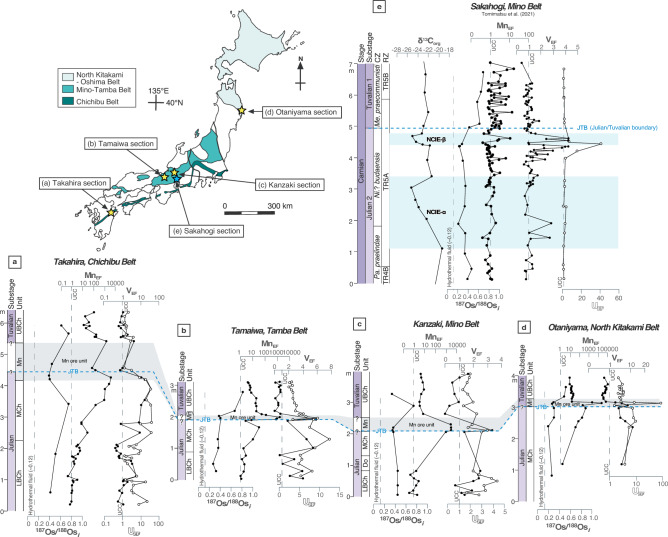
Geochemical data for the Carnian in the Panthalassa. (**a**–**e**) Stratigraphic variations in Os isotope data and element enrichment factors (Mn_EF_, V_EF_, and U_EF_) for pelagic deep-sea sections in Japan. Dashed lines indicate values for the upper continental crust (UCC)^42^ and enrichment values of one. Stratigraphic heights and the biostratigraphy of the studied sections are from Tomimatsu et al.^13^, and for the Sakahogi section is from Tomimatsu et al.^16^. Abbreviations: CZ = Conodont zone, RZ = Radiolarian zone, NCIE = Negative carbon isotope excursion, *Pa*. = *Paragondolella*, *Ni*.? = *Nicoraella*?, *Me. = Metapolygnathus*. The map is created using ACD Systems Canvas Draw software (Version 6.0) (https://www.poladigital.co.jp/canvas/index.html).

The studied sections can be divided into four stratigraphic units (in ascending order): the lower bedded chert (LBCh), massive chert (MCh), Mn ore (Mn), and upper bedded chert (UBCh) units (Supplementary Fig. S2). Ninety-four samples of chert and 19 samples of Mn ore were collected from these stratigraphic units for whole-rock geochemical analysis (Fig. 2). Rhenium and Os concentrations and Os isotope ratios were determined by the isotope dilution method using a multi-collector–inductively coupled plasma–mass spectrometer (MC–ICP–MS; Thermo Fisher Scientific NEPTUNE and NEPTUNE Plus) at the Japan Agency for Marine-Earth Science and Technology (JAMSTEC), Yokosuka, Japan (Supplementary Table S1). Concentrations of major elements and V, Ni, Cu, and Zn were determined with an energy dispersive X-ray fluorescence (XRF) spectrometer using a PANalytical Epsilon 3^XLE^ instrument with a Mo X-ray tube at Kyushu University, Fukuoka, Japan. Trace element analysis was conducted with an ICP–quadrupole mass spectrometer (ICP–QMS; Agilent 7500ce) at the Japan Agency for Marine-Earth Science and Technology (JAMSTEC). The chert and Mn ore samples contain > 80 wt% SiO_2_, meaning that a large amount of biogenic silica dilutes the other major and trace elements (Supplementary Table S2). To avoid the significant dilution effect of biogenic SiO_2_, element concentrations were normalized to Al concentrations and compared with those of upper continental crust (UCC)^42^ to obtain enrichment factors: X_EF_ = (X_sample_/Al_sample_)/(X_UCC_/Al_UCC_), where X and Al are the weight concentrations of elements X and Al, respectively. Details of the analytical protocols are given in the “Methods” section.

## Results

Figure 2 shows the stratigraphic variations in Re and Os concentrations and initial Os isotope ratios (^187^Os/^188^Os_*i*_) in the studied sections. The ^187^Os/^188^Os_*i*_ ratios increase from unradiogenic isotope ratios throughout the Carnian in the LBCh to UBCh units. The Julian (lower Carnian) samples from the stratigraphic interval from the LBCh to Mn units are characterized by relatively uniform and unradiogenic ^187^Os/^188^Os_*i*_ values (Fig. 2): 0.393–0.748 (average = 0.487) in the Takahira section; 0.268–0.411 (average = 0.351) in the Tamaiwa section; 0.349–0.448 (average = 0.402) in the Kanzaki section; and 0.197–0.308 (average = 0.270) in the Otaniyama section ^187^Os/^188^Os_*i*_ values of the UBCh units during the Tuvalian 1 (lowermost Carnian)^13^ are higher than those of the Mn, MCh, and LBCh units (Supplementary Table S1).

Enrichment factors for Mn, Ni, Cu, Zn, V, U, and Mo have been widely used to characterize the redox conditions of marine environments^43–47^, which can be generally classified as oxic, suboxic, anoxic, or euxinic (i.e., presence of free H_2_S)^48^. The Mn_EF_ values are low in the LBCh unit, and generally close to 1 (Fig. 2). The Mn_EF_ values are high in the upper part of the MCh unit and in the Mn unit. Samples from the UBCh unit have much higher values of Mn_EF_ than those from the LBCh unit in all studied sections. The V_EF_ values of each studied section are much higher in the stratigraphic interval between the LBCh and MCh units (Fig. 2). The U_EF_ values are also high in these units, similar to the variations in V_EF_. The V_EF_ and U_EF_ values decrease gradually across the JTB, reaching low values in the UBCh unit. A similar pattern of decreasing V_EF_ and U_EF_ with increasing Mn_EF_ has also been reported from a Toarcian bedded chert sequence in the Mino Belt, central Japan^49^. The stratigraphic variations in Ni_EF_, Cu_EF_, Zn_EF_, and Mo_EF_ are similar to those in V_EF_ and U_EF_ in the studied sections (Supplementary Table S2). Although some samples in the Mn units exhibit Mo enrichments (Mo_EF_ < 100), likely due to scavenging by Mn oxides^50^, almost all samples have Mo concentrations and Mo_EF_ values that are lower than or similar to UCC values.

**Figure 3:**
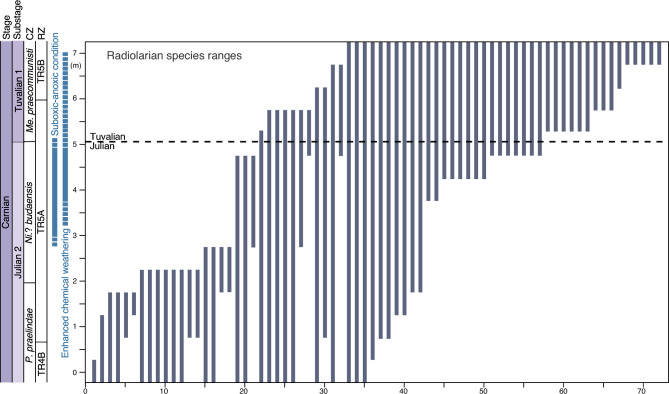
Stratigraphic ranges of Carnian radiolarian species across the JTB. Species numbers are shown on the *x*-axis. Supplementary Table S3 explains the radiolarian taxon ranges. Abbreviations: CZ = Conodont zone, RZ = Radiolarian zone, *Pa*. = *Paragondolella*, *Ni*.? = *Nicoraella*?, *Me. = Metapolygnathus.*

We analyzed a large dataset of Carnian conodont and radiolarian occurrences from the studied sections^13, 16, 51, 52^ to assess the magnitude of the extinction related to the CPE. In the uppermost Julian, most of the conodont species disappear, including all species belonging to genera *Nicoraella*?*, Gladigondolella*, and *Paragondolella*, except for *Paragondolella polygnathiformis* and *Paragondolella praelindae*. Species richness of the conodonts does not increase during the Tuvalian 1 in the studied sections. However, in the higher stratigraphic levels of the Sakahogi section, diversity increased in the upper Tuvalian (Tuvalian 2 and 3), with the appearance of several species belonging to the genera *Carnepigondolella, Metapolygnathus*, *Kraussodontus*, *Primatella*, *Norigondolella*, *Hayashiella*, and *Quadralella*^16, 52^. Furthermore, investigation of the stratigraphic ranges of the radiolarian species indicates a steady decrease in the diversity of Julian species throughout the late Julian to Tuvalian (Fig. 3), with an abrupt increase in diversity of Tuvalian species across the JTB (Fig. 3).

## Discussion

**Figure 4:**
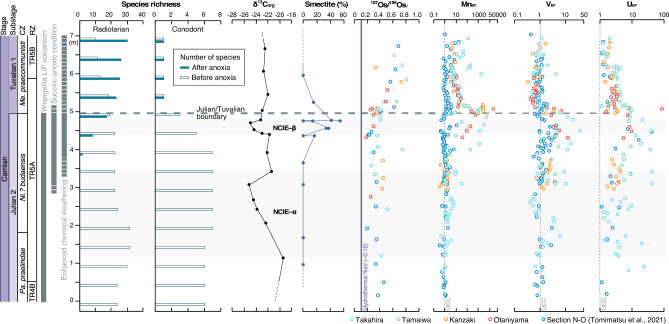
Geochemical and biotic changes in the Panthalassa during the CPE. Faunal compositions of conodont and radiolarian species, and chemostratigraphic records of δ^13^C_org_, ^187^Os/^188^Os_*i*_ (t = 230 Ma), and enrichment factors of redox-sensitive elements (Mn_EF_, V_EF_, and U_EF_) in the studied sections projected onto a composite section for the Carnian. The occurrence of smectite is based on Nakada et al.^12^. The radiolarian and conodont biozones are based on Tomimatsu et al.^13, 16^ and Sugiyama^51^. Abbreviations: CZ = Conodont zone, RZ = Radiolarian zone, NCIE = Negative carbon isotope excursion, *Pa*. = *Paragondolella*, *Ni*.? = *Nicoraella*?, *Me. = Metapolygnathus.*

In all the studied sections, the ^187^Os/^188^Os_*i*_ values are unradiogenic and uniform during the Julian (Fig. 4). A large input of mantle-derived unradiogenic Os during emplacement of the Wrangellia LIP has been proposed to explain the unradiogenic ^187^Os/^188^Os_*i*_ ratios during the Julian^16^. The studied sections exhibit the most unradiogenic ^187^Os/^188^Os_*i*_ ratios in the latest Julian. This indicates that the Wrangellia LIP volcanism peaked at that time. A recent Hg isotope study also provides evidence that the Wrangellia LIP volcanism peaked in the latest Julian, which is documented by a negative shift in Δ^199^Hg values of the Sakahogi section^20^. Across the JTB, there was an abrupt increase in ^187^Os/^188^Os_*i*_ values, followed by radiogenic values of 0.5–0.8 during the Tuvalian. There is also an increase in the relative abundance of smectite^12^ at the onset of the increase in ^187^Os/^188^Os_*i*_ values in the JTB section, which is a lateral extension of the bedded chert exposed in the Sakahogi section (Fig. 4). The presence of smectite across the JTB has been interpreted as evidence of continental humidification. Therefore, we interpret that the abrupt increase in ^187^Os/^188^Os_*i*_ across the JTB we observed may reflect a massive input of radiogenic Os due to enhanced continental weathering associated with the humid climate in Pangea, combined with the weakened supply of unradiogenic Os associated with the Wrangellia volcanism.

The synchronous increase in V_EF_ and U_EF_ values suggests that suboxic to anoxic conditions were temporarily widespread in the pelagic deep-sea Panthalassa during the late Julian (Fig. 4). However, the enrichment factor of Mo is consistently low during the periods of V and U enrichment, suggesting that the deep-sea environment was not euxinic. Late Julian marine anoxia has been identified based on the widespread deposition of black shales and organic-rich marls in Tethyan marginal basins (e.g., Northern Calcareous Alps^6^, South China^10, 11^, Sicily^53^, Dolomites, Southern Alps^54^, and Tunisia^55^). This suggests that marine anoxia may have developed from the shallow continental margin into the equatorial deep-sea Panthalassa basin (Supplementary Fig. S3). Increased continental weathering and nutrient flux during the CPE have been proposed as a possible trigger for the ocean anoxia^6, 27, 28^. However, ^187^Os/^188^Os_*i*_ ratios increased abruptly after this period of suboxic to anoxic conditions, which argues against a link between increased chemical weathering and the development of suboxic to anoxic conditions in the Panthalassa. A simple explanation is that the marine anoxia occurred as a result of oceanic stagnation and stratification, as proposed for the Rhaetian of the Late Triassic^56^.

The Triassic was one of the warmest periods in the Phanerozoic, with ice-free poles^57^. Ocean circulation would not have been as strong as today, given the absence of cold polar waters. Higher temperatures at high latitudes, where bottom waters form, would have reduced the solubility of oxygen, making the deep ocean more susceptible to anoxic conditions. Therefore, the weakening of oceanic circulation during a global warming event might explain the formation of a relatively stagnant ocean that led to the development of oxygen-depleted conditions in deep-sea Panthalassa during the late Julian. Such temperature increases during the late Julian are supported by a decrease in conodont apatite oxygen isotope composition (δ^18^O_phos_) across the JTB in the marine successions of the Tethyan realm (Supplementary Fig. S4). In the eastern Tethys, South China Brock, the δ^18^O_phos_ record indicates two pulse warming events in the late Julian and Tuvalian^10^. In the western Tethys, the temperature estimates from δ^18^O_phos_ from the Northern Calcareous Alps and the Lagonegro Basin show a single pulse warming from the late Julian to early Tuvalian^6, 7, 9^. Although there are differences in the trend of the data from each section, their results suggest that the δ^18^O_phos_ records from the Tethyan realm shows decreasing trends, indicating global warming during the late Julian to early Tuvalian (Supplementary Fig. S4).

The Mn_EF_ values increase as the V_EF_ and U_EF_ values decrease and reach a peak at the JTB. These geochemical trends suggest that oxygen-depleted conditions were temporarily widespread in the deep pelagic Panthalassa during the late Julian, but then recovered rapidly to oxic conditions associated with Mn precipitation. The recovery to an oxic ocean coincided with the onset of an increase in continental weathering associated with the humid climate of Pangea, as indicated by the increase in the Os isotope ratios and the occurrence of abundant smectite (Fig. 4). Acceleration of the hydrological cycle may have played an important role in the recovery from suboxic to anoxia in the Panthalassa. Notably, Mn ore deposits were only formed on the younger oceanic-plate sediments of the Panthalassa^13^ during the recovery to an oxic ocean. Younger oceanic-plate sediments in the Jurassic accretionary complexes of Japan may have been deposited in the vicinity of the Wrangellia LIP, because they were adjacent to the oceanic plate that records the eruption of the Carnian oceanic basalts (Fig. 1). As such, the Mn ore deposits with low ^187^Os/^188^Os_*i*_ values in the studied sections may have formed from Mn released by Wrangellia LIP volcanism.

Although the precise cause of the extinction of pelagic fauna at the JTB is controversial, recent studies have shown that most lower Carnian conodont taxa disappeared across the JTB with the lowest diversity at the lowermost Tuvalian^4, 26^. Zhang et al.^58^ also reported that the loss of conodont diversity and abundance occurred in association with marine anoxia and temperature fluctuations, based on a study of a section in southwestern China. In the Panthalassa Ocean, extinction of typical Julian conodont fauna, such as *Gladigondolella* spp., *Paragondolella inclinata*, *Paragondolella foliata*, and *Nicoraella*? *budaensis*, occurred at the end of the Julian, and the low species diversity persists above the JTB (Fig. 4). It has been suggested that *Gladigondolella* inhabits cooler, deeper waters, which is suggested by the consistently higher δ^18^O_phos_ values^9^. These biostratigraphic data suggest that extinction of Julian conodonts, including cooler deep-water taxa, in the Tethys and Panthalassa oceans was associated with the peak interval of marine anoxia. The number of radiolarian species characteristic of the upper Ladinian and Julian decreased from 22 to 18 species across the late Julian marine anoxia event (Fig. 4). In contrast, the radiolarian diversity of the Tuvalian taxa increased significantly after the anoxia to 30 species in the early Tuvalian. These results suggest that radiolarian species in the Panthalassa were less affected by marine anoxia. Further research is needed to understand the habitat (i.e., water depth and temperature) of Carnian conodonts and radiolarians in the Panthalassa to clarify the relationship between marine anoxia and selective conodont and radiolarian extinction, but in addition to anoxia, ocean acidification and eustatic sea-level fall have been suggested to have occurred during the CPE^5, 59^, both of which have significant impacts on marine ecosystems. Ocean acidification and sea-level fall have been implicated as causes of the Toarcian (Early Jurassic) and end Cretaceous extinctions^60–62^, but radiolarians were not affected in either case^63, 64^. Although ocean acidification and sea-level fall during the CPE could potentially explain the selectivity between conodont and radiolarian extinctions, it is difficult to determine the cause of the extinction selectivity at present. However, the increased radiolarian diversity during the Tuvalian may have resulted from increased nutrient inputs and more abundant food sources, such as dinoflagellates and coccolithophores in the ocean^27^. Global warming associated with Wrangellia LIP volcanism^6, 28^ possibly contributed to increased productivity through enhanced chemical weathering associated with the humid climate of Pangea and a greater flux of nutrients (e.g., Fe-bearing clay minerals^12^) into the ocean.

## Conclusions

We investigated the Carnian marine environment as recorded in five bedded chert successions in Japan, based on stratigraphic variations of marine Os isotope data, redox-sensitive elements (e.g., Mn, V, and U), and conodont and radiolarian biostratigraphy. The Os isotope ratios are unradiogenic in the Julian, reaching the most unradiogenic in the latest Julian, and increase upwards across the JTB. This Os isotope trend indicates that Wrangellia LIP volcanism peaked at the end-Julian, resulting in significant input of unradiogenic Os into the Panthalassa Ocean. Redox-sensitive elements indicate that suboxic to anoxic conditions existed in the Panthalassa in the latest Julian, coinciding with the peak of Wrangellia LIP volcanism. Our biostratigraphic analysis reveals that conodont extinction coincided with the peak interval of oceanic anoxia. In contrast, radiolarian species were not severely affected by the marine anoxia in the Panthalassa. The diversification of radiolarians began after the JTB, probably associated with accelerated chemical weathering and increased nutrient fluxes to the ocean associated with global warming and the humid climate of Pangea.

## Methods

### Sample preparation for geochemical analysis

The collected samples were crushed and handpicked to avoid contamination from veins and strongly recrystallized/weathered materials. Samples were washed in Milli-Q deionized water by ultrasonic cleaning (> 18.2 MΩ cm). Samples for major element (94 chert samples and 19 Mn ore samples) and trace element (94 chert and 19 Mn ore samples) analyses were finely crushed using a Multi-Beads Shocker (PV1001; Yasui Kikai, Japan), and samples for Re–Os isotope analysis (32 chert and 8 Mn ore samples) were powdered in an agate mortar–ball mill.

### Rhenium and Os isotope analysis

Concentrations and isotope ratios of Re and Os were determined by the isotope dilution method using an MC–ICP–MS (Thermo Fisher Scientific Neptune and Neptune Plus) at JAMSTEC, Yokosuka, Japan. Approximately 2.0 or 2.5 g of powdered Mn ore and chert samples were weighed, spiked with ^185^Re and ^190^Os, and digested in 4 mL of inverse aqua regia in a Carius tube at 220 °C for 24 h. After cooling, the Carius tube was opened, and the solution was transferred to a Teflon vial, along with 9 mL of Milli-Q deionized water. Johnson Matthey Company (JMC) Os standard solutions (^187^Os/^188^Os = 0.106838 ± 0.000015)^65^ containing 40 pg of total Os were also prepared and processed in each analytical batch to assess the data accuracy. The Os isotope ratios were determined by MC–ICP–MS combined with the sparging introduction of OsO_4_ gas molecules into the ICP glass torch^65–68^, using Faraday cups (FCs) for manganese ore samples and compact discrete dynode (CDD) multi-ion-counting detectors for blank and chert samples. Different yields among three CDDs was corrected by in-run cross-calibration by monitoring ^190^Os with a quadratic drift correction^68^ and instrumental mass bias within the MC–ICP–MS was internally corrected by normalizing with ^192^Os/^188^Os = 3.08271 (Ref.^69^) when using both CDDs and FCs. The typical Ar gas flow rate during the Os isotope measurement was 1.2 L min^−1^, which is 40 to 50 times larger than the volume of the Teflon vials containing the sample and standard solutions. Thus, the memory effect due to the previous sample was quickly removed by the Ar gas flushing with less than 30 s. In this case, the memory effect due to analysis of a previous sample was quickly removed by Ar gas flushing for < 30 s. After the Os isotope measurement, the sample solutions were evaporated on a hotplate at 140 °C to remove the remaining Os. Rhenium was then purified using a two-step column separation procedure with anion exchange resin (Muromac AG 1-X8) following the methods of Nozaki et al.^65^, Ohta et al.^68^, and Morgan et al.^70^. Rhenium isotope ratios were measured by MC–ICP–MS with solution introduction into the glass torch, using the Ir standard addition method for external mass bias correction by using ^193^Ir/^191^Ir = 1.68097 (Refs.^68, 71^). The Re blank was 0.44 pg, and the Os blanks were 0.12 and 0.09 pg, with ^187^Os/^188^Os ratios of 0.157 and 0.221, respectively.

The initial ^187^Os/^188^Os ratios of the chert and Mn ore samples were calculated using the sample ages estimated based on the biostratigraphy^13^ with the ^187^Re decay constant of 1.666 × 10^–11^ year^–1^ (Ref.^72^) and the astronomically tuned geomagnetic polarity timescale. We used an age of 230 Ma to correct all the Os isotope data. Although the depositional ages of the chert successions in the four studied sections range in age from the base of the Carnian (ca. 237.0 Ma) to the end-Carnian (ca. 221.0 or 228.4 Ma), the differences in ^187^Os/^188^Os_*i*_ values between 237.0 and 221.0 Ma (or 228.4 Ma) are typically < 0.01 (< 2%) due to the low ^187^Re/^188^Os ratios (< 33) of the chert and Mn ore samples. Therefore, the uncertainty associated with the age of the sediments is negligible.

### Major and trace element analysis

Concentrations of major elements (Si, Ti, Al, Fe, Mn, Mg, Ca, Na, K, and P) and V, Ni, Cu, and Zn were determined with an energy dispersive X-ray fluorescence (ED-XRF) spectrometry using a PANalytical Epsilon 3^XLE^ instrument with a Mo X-ray tube at Kyushu University. Powdered samples were oven-dried at 110 °C for 2 h and then mixed with a binder (cellulose) at a ratio of 5:1 (2.0 g of sample to 0.4 g of binder). The mixture was finely homogenized using a Multi-Beads Shocker and pressed at 20 t for 3 min to form a pressed powder pellet. Analyses were calibrated using 22 standard rock samples issued by the Geological Survey of Japan. The detection limits for trace elements were 9 ppm for V, 3 ppm for Ni, and 2 ppm for Cu and Zn. Reproducibility based on replicate analysis of two standards (JSd-1 and JCh-1) was better than ± 1% for Al, Mn, Na, V, Ni, Cu, and Zn; ± 3% for Ti, Ca, and K; and ± 10% for P.

Trace element analysis was conducted with an ICP–QMS (Agilent 7500ce) at JAMSTEC. 50 mg of powdered samples were dissolved in a mixture of HNO_3_–HClO_4_–HF in Teflon vials, and then heated on a hotplate at 110 °C overnight. The digested samples were progressively evaporated at 110 °C for 12 h, 130 °C for 3 h, and 160 °C until dryness. Then, the remaining sample solution (several drops) was dissolved again into 4 mL of 68% m/m HNO_3_, 1 mL of 30% m/m HCl, and 5 mL of Milli-Q deionized water, and then heated at 110 °C, followed further diluted to 1:100 by mass (total dilution factor ~ 20,000) before introduction into the ICP-QMS instrument. Details of the ICP–QMS analytical procedures were described by Takaya et al.^73^.

### Supplementary Information

Supplementary Information 1.

Supplementary Table S1.

Supplementary Table S2.

## Data Availability

All data generated or analyzed during this study are included in this published article and supplementary information files.
