# Supplementary material for: Pelagic responses to oceanic anoxia during the Carnian Pluvial Episode (Late Triassic) in Panthalassa Ocean
Source: Sci Rep. 2023 Sep 28;13:16316. doi: 10.1038/s41598-023-43525-9 (PMC10539534; doi:10.1038/s41598-023-43525-9)
Supplement: Supplementary file 1 — Supplementary Information 1. [file 41598_2023_43525_MOESM1_ESM.docx]

Tomimatsu et al. 2023

**Supplementary Information**

**Pelagic responses to oceanic anoxia during the Carnian Pluvial Episode (Late Triassic) in Panthalassa Ocean**

Yuki Tomimatsu^1*^, Tatsuo Nozaki^2,3,4^, Tetsuji Onoue^1^, Hironao Matsumoto^2^, Honami Sato^1^, Yutaro Takaya,^2,5,6^, Jun-Ichi Kimura^7^, Qing Chang^7^, Manuel Rigo^8,9^

^1^ *Department of Earth and Planetary Sciences, Kyushu University, Fukuoka 819-0395, Japan*

^2^ *Submarine Resources Research Center, Research Institute for Marine Resources Utilization, Japan Agency for Marine-Earth Science and Technology (JAMSTEC), Kanagawa 237-0061, Japan*

^3^ *Frontier Research Center for Energy and Resources, The University of Tokyo, Tokyo 113-8656, Japan*

^4^ *Department of Planetology, Kobe University, Hyogo 657-8501, Japan*

^5^ *Department of Systems Innovation, The University of Tokyo, Tokyo 113-8656, Japan*

^6^ *Faculty of Science and Engineering, Waseda University, Tokyo 169-8555, Japan*

^7^ *Volcanoes and Earth’s Interior Research Center, Research Institute for Marine Geodynamics, Japan Agency for Marine-Earth Science and Technology (JAMSTEC), Kanagawa 237-0061, Japan*

^8^ *Department of Geosciences, University of Padova, Padova 35131, Italy*

^9^ *Institute of Geosciences and Earth Resource (IGG–CNR), Padova 35131, Italy*

*Corresponding author

*E–mail address*: tomimatsu.yuki.396@m.kyushu-u.ac.jp (Y. Tomimatsu)

This PDF file includes:

Supplementary Figures S1–S3

Supplementary Table S3

**Supplemental Material**

**Stratigraphy of the study sections**

***Takahira section***

The Takahira section is located in the Shakumasan Group of the Chichibu Belt in the Saiki area of western Kyushu (Supplementary Fig. S1a). The Chichibu Belt is a Jurassic subduction-generated accretionary complex that extends northeastward in the Southwest Japan^1^. The Shakumasan Group consists of a coherent sequence of Lower Triassic to Middle Jurassic bedded cherts (Enoura Formation) and overlying Middle Jurassic terrigenous clastic rocks (Youra Formation)^2^. The bedded cherts of the Enoura Formation range in color from dark red to light green, dark gray, and black to white. They occur as extensive beds and can be traced laterally for several kilometers. Since the bedded cherts contain no carbonate and coarse terrigenous grains, they are interpreted to have been deposited in a pelagic deep-sea setting deeper than the carbonate compensation depth (CCD) and away from terrestrial areas^1-4^. Based on paleomagnetic data, the Triassic bedded cherts of the Enoura Formation are considered to be deep-sea sediments that accumulated in an open-ocean environment within a low-latitude zone of the Panthalassa Ocean^5^.

The Takahira section (Global Positioning System coordinates: 33◦00′18′′N, 131◦48′06′′E) consists of an ascending sequence of the lower bedded chert unit, the massive chert unit containing “Akashiro” silica stone, the manganese ore unit, and the upper bedded chert unit (aka means red and shiro means white in Japanese, respectively)^6,7^. The bedded cherts comprise a series of rhythmic couplets of thin shale beds (<3 mm thick) intercalated with 3-10 cm thick chert beds. The light gray to dark gray and reddish to purple color of the lower and upper chert units, respectively. This silica stone is over 1.5 m thick and consists of reddish chert breccia infilled with a quartz matrix that does not contain microfossils. The bedded manganese layers are dark gray to black in color. They are thickly bedded (5-13 cm thick) with few intercalated mudstone layers. The manganese ores in the Takahira deposit consist mainly of rhodochrosite and manganese oxide minerals^8^.

The lower and upper bedded chert and manganese ore units contain abundant radiolarian fossils. The stratigraphic interval from the lower bedded chert unit to the manganese ore unit correlates with the TR5A (*Capnuchosphaera*) Zone, and the upper bedded chert unit corresponds to the TR5B (*Poulpus carcharus*) Zone^6,7^ (Supplementary Fig. S2). The TR5A and TR5B zones are calibrated based on the co-occurrence of conodont species from the Inuyama area^9,10^. Based on the conodont data, the TR5A and TR5B zones correlate from late early Carnian (Julian) to early late Carnian (Tuvalian) and late Carnian (Tuvalian), respectively (Supplementary Fig. S2). The stratigraphic interval from the lower bedded chert unit to the manganese ore unit correlates with the *Mazzaella carnica* – *Paragondolella praelindae* zones, assigning the late Julian to the latest Julian^7^. The biostratigraphic studies of the conodonts suggest that the stratigraphic interval between the manganese ore unit and the lower part of the upper bedded chert unit can be correlated with the Julian-Tuvalian boundary.

***Tamaiwa section***

The Tamaiwa section is located in the Tamba Belt in the Yotsuya area of central Japan. The Tamba Belt has been divided into the structural lower part, "Type 1 Suite", and the structural upper part, "Type 2 Suite"^11^. The Type 1 suite is widely exposed in the Yotsuya area, which is divided into three tectonic complexes (Tsurugaoka, Sasae and Yuragawa complexes) based on lithology, age, and internal geologic structure^12-14^. The studied section is part of the Yuragawa Complex (Supplementary Fig. S1b). The Yuragawa Complex consists of tectonic slabs of Triassic to Upper Jurassic siliceous rocks (siliceous claystone and chert) with pelitic mixed rocks^12^. The slabs are laterally continuous and tens of kilometers long. They consist of a sequence of siliceous claystone, chert, siliceous mudstone, mudstone and sandstone in ascending stratigraphic order. The Yuragawa Complex is considered to be the upper part of the original sequence on the Panthalassic oceanic plate^14^.

The Tamaiwa section (GPS coordinates: 35◦13′49′′N, 135◦32′40′′E) contains the lower bedded chert unit, the massive chert unit, the manganese ore unit, and the upper bedded chert unit, in ascending order. These bedded cherts consist of rhythmic alternations of chert (1–6 cm thick) and shale beds (0.3–3 cm thick). The total thickness of the massive chert unit is approximately 80 cm. The bedded chert and massive chert are light to dark gray in color. The manganese ore unit is black in color and about 20 cm thick.

The radiolarian assemblage of the upper bedded chert unit is comparable to the TR 5B Zone^7^ (Supplementary Fig. S2). Conodont fossils are common in the bedded and massive cherts throughout the Tamaiwa section. The stratigraphic interval from the bedded chert unit to the massive chert unit correlates with the *Paragondolella praelindae* zone assigned to the late Julian age^7^. The upper bedded chert unit corresponds to the *Metapoylgnathus praecommunisti* zone, which indicates an early Tuvalian age^7^. The biostratigraphic studies of the conodonts suggest that the stratigraphic interval between the upper massive chert unit and the lower part of the upper bedded chert unit can be correlated with the Julian-Tuvalian boundary (Supplementary Fig. S2).

***Kanzaki section***

The Kanzaki section is located in the Mino Belt in the Tanigumi area, central Japan (Supplementary Fig. S1c). The Mino Belt is divided into six tectonostratigraphic units based on lithology, structure, and age of accretion^15^: the Sakamoto-toge, Samondake, Funabuseyama, Nabi, Kamiaso, and Kanayama units. The studied section belongs to the Kanayama unit. This unit consists of mélanges and is composed of abundant blocks and sheets of various sizes of pelagic bedded chert, hemipelagic siliceous mudstone, and sandstone with minor amounts of greenstone and limestone in a weakly sheared shale matrix^15^. The larger sheets of siliceous rocks (chert, siliceous claystone, and siliceous mudstone) preserve the original Ocean Plate Stratigraphy (OPS)^15,16^. The OPS reconstructed from Katsuyama Unit consists of Lower Triassic siliceous claystone, Middle Triassic–Lower Jurassic chert, and Middle–Upper Jurassic siliceous mudstone in ascending order (Wakita, 1988)^15^. Radiolarian ages of the shale matrix indicate Late Jurassic to earliest Cretaceous, suggesting that the Kanayama unit is the youngest accretionary unit in the Mino Belt^15,17^.

The Kanzaki section (GPS coordinates: 35◦37′24′′N, 136◦42′6′′E) includes the lower bedded chert unit, the massive chert unit with a dolomite layer at the base, the manganese ore unit, and the upper bedded chert unit, in ascending order. The upper and lower bedded chert units are light gray to gray in color. They consist of rhythmically alternating layers of chert (1–6 cm thick) and relatively thin layers of shale (< 3 mm). The thick dolomite layer lies between the lower bedded chert unit and the massive chert unit. A few layers of dolomite, several centimeters thick, are intercalated within the basal part of the upper bedded chert unit. Weathered dolomite surfaces are reddish-brown, while freshly fractured surfaces are greenish-gray. The dolomite layers are fine-grained and mostly euhedral crystals. The massive white to gray chert unit approximately 80 cm thick underlies the manganese ore unit. The manganese ore unit is dark gray to black in color, weakly bedded, and approximately 40 cm thick. Previous studies have not reported the manganese-bearing minerals in the Kanzaki section.

Tomimatsu et al.^7^ recognized the TR5A (*Capnuchosphaera*) radiolarian biozone in the lower bedded chert unit of the Kanzaki section, which probably correlates with the Julian age (Supplementary Fig. S2). Based on their conodont data, three conodont zones are recognized in the upper bedded chert unit: the *Hayashiella tuvalica*, the *Metapolygnathus praecommunisti*, and the *Carnepigondolella orchardi* zones^7^ (Supplementary Fig. S2). The upper bedded chert unit is correlated with Tuvalian age. Considering the biostratigraphic evidence of radiolarians and conodonts, the stratigraphic interval between the lower bedded and upper bedded chert units probably correlates with the Julian-Tuvalian boundary.

***Otaniyama section***

The Otaniyama section is located in the North Kitakami Belt, ~20 km southwest of Miyako City, northeastern Iwate, Japan (Supplementary Fig. S1d). The North Kitakami Belt is divided by the Seki-Odaira Fault (or Iwaizumi Tectonic Lime) into the Kuzumaki–Kamaishi and Akka–Tanohata sub-belts. The North Kitakami Belt is interpreted as a northern extension of the Chichibu Belt and extends to the Oshima Belt in southwestern Hokkaido. The Otaniyama section belongs to the Kuzumaki–Kamaishi Sub-belt. This sub-belt consists of upper Carboniferous–lower Permian and Middle to Upper Triassic sequences of basaltic rocks, limestone, and bedded chert, upper Carboniferous–Middle Jurassic pelagic chert, Middle–Upper Jurassic hemipelagic siliceous mudstone, and Middle–Upper Jurassic terrigenous rocks^18^.

The Otaniyama section (GPS coordinates: 39◦29′10′′N, 141◦50′32′′E) is composed of the massive chert unit, the manganese ore unit, and the upper bedded chert unit, in ascending order. The massive chert unit is >3.0 m thick and divided into a lower and an upper part. The lower part of the massive chert unit (0 to 2.5 m) is light gray. The upper part of the massive chert unit (2.6 to 3.0 m) is variable in color from dark brown to red, with the "Akashiro" silica stone present at the base. The manganese ore (25–60 cm thick) occurs conformably within the chert sequence, which varies in color from dark brown, dark red, grayish green, and yellowish brown to pink. The manganese-bearing minerals from Otaniyama deposit recognized in this section are tephroite, rhodonite, penwithite, rhodochrosite, jacobsite, and braunite^19,20^. The upper bedded chert unit is reddish to purplish in color and consists of alternating thick chert beds (2–20 cm thick) with few intercalated shale beds.

The upper bedded chert and massive chert units of the Otaniyama section rarely yield conodonts and poorly preserved radiolarian fossils. Okami^21^ reported the occurrence of Late Triassic (Carnian to Norian) conodont fossils from the chert associated with manganese ore at the Otaniyama deposit. It has recently been reported that the upper part of the massive chert unit has a stratigraphic position of upper Julian to lower Tuvalian, based on the conodont species (*Nicoraella*? sp.) yielded by the upper part of the massive chert unit^7^.

**Supplemental Figures:**

**Supplementary Fig. S1.** Geological maps of (a) the Saiki area in the Chichibu Belt (Nishi^2^), (b) the Yotsuya area in the Tamba Belt (Kimura et al.^12^), (c) the Tanigumi area in the Mino Belt (Wakita^16^), (d) the southwestern area of Miyako City in the North Kitakami Belt (Yoshida et al.^20^). These maps are created using ACD Systems Canvas Draw software (Version 6.0) (https://www.poladigital.co.jp/canvas/index.html).

**Supplementary Fig. S2.** Stratigraphic distribution of radiolarian and conodont fossils in the Takahira, Tamaiwa, Kanzaki, and Otaniyama sections. The sections are mainly divided into four stratigraphic units: the lower bedded chert unit (LBCh) with the dolomite layer (Do) at the top; the massive chert unit (MCh); the manganese ore unit (Mn); and the upper bedded chert unit (UBCh), in ascending stratigraphic order. Abbreviations: *Pa.* = *Paragondolella*, *Ma*. = *Mazzaella*, *Me.* = *Metapolygnathus*, *Ca.* = *Carnepigondolella*, and *P.* = *Poulpus*.

**Supplementary Fig. S3.** Comparing the profiles of enrichment factors of Vanadium and Uranium from the Carnian successions in Panthalassa (Tomimatsu et al.,^10^; This study) and Eastern Tethys (Sun et al.,^22^; Zhang et al.,^23^). Abbreviations: CZ = Conodont zone; RZ = Radiolarian zone. For the stratigraphic units of the study sections, refer to Supplementary Fig. S2.

**Supplementary Fig. S4.** Organic carbon isotope records from the pelagic deep-sea Panthalassa (this study), South China (Sun et al.^22^), and NW Tethys (Dal Corso et al.^24-26^), compared with initial ^187^Os/^188^Os ratios (^187^Os/^188^Os*_i_*) from the study section. Paleotemperatures calculated from oxygen isotope compositions of conodont apatite (δ^18^O_phos_) from South China (Sun et al.^22^), Northern Calcareous Alps (NCA; Hornung et al.^27^) and Lagonegro Basin (LA; Rigo and Joachimski^28^; Trotter et al.^29^) are also shown for comparison. Green and blue of lines indicate the data for oxygen-isotope compositions of conodont apatite determined by high temperature conversion elemental analyzer (TC–EA) and sensitive high-resolution ion microprobe (SHRIMP), respectively. Abbreviations: CZ = Conodont zone; NCIE = Negative carbon isotope excursion; V-SMOW = Vienna standard mean ocean water.

**Supplementary Table S3.** List of radiolarian species. Explanation of the stratigraphic ranges of radiolarian species, from left to right, as displayed in Fig. 3.

1. *Pseudostylosphaera goestlingensis* (Kozur and Mostler, 1979)
2. *Praeheliosaturnalis leavis* Kozur and Mostler, 1972
3. *Muelleritortis* sp. A
4. *Tritortis kretaensis* (Kozur and Krahl, 1984)
5. *Veles* sp. A
6. *Planispinocyrtis multiporata* Kozur and Mostler, 1994
7. *Canesium* (?) *cucurbita* Sugiyama, 1997
8. *Corum* sp. A Sugiyama, 1997
9. *Muelleritoritis cive* Sugiyama, 1997
10. *Triassocampe nodosoannulata* (Kozur and Mostler, 1994)
11. *Pseudostylosphaera gracilis* Kozur and Mock, 1981
12. *Vinassaspongus subsphaericus* Kozur and Mostler, 1979
13. *Pentaspongodiscus mesotriassicus* Dumitrica et al., 1980
14. *Tritortis* sp. A
15. *Hindeosphaera bispinosa* (Kozur and Mostler, 1979)
16. *Tricolocapsa* (?) *lacrima* Sugiyama, 1997
17. *Deflandrecyrtium* sp. A
18. *Elbistanium gracie* Tekin and Bedi, 2007
19. *Falcispongus curvispinosus* (Domitrica, 1982)
20. *Hungarosaturnalis* sp. A
21. *Heliosaturnalis* sp. cf. *H. transitus* Kozur and Mostler, 1979
22. *Pseudostylosphaera japonica* (Nakaseko and Nishimura, 1979)
23. *Archaeosemantis pterostephanus* Dumitrica, 1978
24. *Paronaella trammeri* (Kozur and Mostler, 1978)
25. *Pseudostylosphaera nazarovi* (Kozur and Mostler, 1979)
26. *Triassocampe* sp. aff. *T. sulovensis* Kozur and Mock, 1981
27. *Xiphothecaella longa* Kozur and Mock, 1981
28. *Xiphothecaella rugosa* Bragin, 1991
29. *Sarla* (?) *extema* Blome, 1983
30. *Poulpus phasmatodes* De Wever, 1979
31. *Pseudostylosphaera longispinosa* Kozur and Mostler, 1981
32. *Praemesosaturnalis* (?) *ormites* Sugiyama, 1997
33. *Archaeosemantis cristianensis* Dumitrica, 1982
34. *Hozmadia* sp. A
35. *Triassocampe postdeweveri* Kozur and Mostler, 1994
36. *Corum* (?) *delgado* Sugiyama, 1997
37. *Pachus* (?) *indistinctus* Blome, 1984
38. *Annulotriassocampe baldii* Kozur, 1994
39. *Poulpus* (?) *coronensis* Yeh, 1990
40. *Cenophaera clathrata* Parona, 1980
41. *Pseudostylosphaera hellenica* (De wever, 1979)
42. *Veles vulgaris* Sugiyama, 1997
43. *Capnuchosphaera triassica* De Wever, 1979
44. *Capnuchosphaera deweveri* Kozur and Mostler, 1979
45. *Laxtorum* (?) *carnicum* Sugiyama, 1997
46. *Archaeosemantis lithocircites* Sugiyama, 1997
47. *Capnuchosphaera sagaris* Sugiyama, 1997
48. *Kahlerosphaera* sp. A
49. *Multimonilis* sp. B
50. *Trialatus pristinus* Sugiyama,1997
51. *Tetraspinocyrtis yehae* (Sugiyama, 1997)
52. *Capnuchosphaera crassa* Yeh, 1990
53. *Kahlerosphaera* sp. B
54. *Neopylentonema procea* Sugiyama, 1997
55. *Poulpus* sp. A
56. *Sarla delicata* Blome, 1983
57. *Xiphothecaella karpenissionensis* De Wever, 1979
58. *Capnuchosphaera colemani* Blome, 1983
59. *Kahlerosphaera norica* Kozur and Mock, 1981
60. *Multimonilis japonicus* Sugiyama, 1997
61. *Poulpus carcharus* Sugiyama, 1997
62. *Syringocapsa batodes* De Wever, 1979
63. *Trialatus longicornutus* Yeh, 1990
64. *Poulpus piabyx* De Wever, 1979
65. *Trialatus megacornutus* Yeh, 1990
66. *Capnuchosphaera tricornis* De Wever, 1979
67. *Palaeosaturnalis* (?) *incomptus* Sugiyama, 1997
68. *Capnuchosphaera contracta* Kozur and Mock, 1981
69. *Capnuchosphaera lea* De Wever, 1979
70. *Capnuchosphaera theloides* De Wever, 1979
71. *Canesium lentum* Blome, 1984
72. *Japonocampe nova* Yao, 1982

References

1. Matsuoka, A., Jurassic–Early Cretaceous tectonic evolution of the Southern Chichibu terrane, southwest Japan. *Palaeogeogr. Palaeoclimatol. Palaeoecol.* **96**, 71–88 (1992).
2. Nishi, T. Geology and tectonics of the Sambosan Terrane in eastern Kyushu, Southwest Japan – Stratigraphy, sedimentological features of the depositional setting of the Shakumasan Group. *J. Geol. Soc. Japan* **100**, 199–215 (1994).
3. Onoue, T., Nakamura, T., Haranosono, T. & Yasuda, C. Composition and accretion rate of fossil micrometeorites recovered in Middle Triassic deep-sea deposits. *Geology* **39**, 567–570 (2011).
4. Muto, S., Takahashi, S., Yamakita, S. & Onoue, T. Scarcity of chert in upper lower Triassic Panthalassic deep-sea successions of Japan records elevated clastic inputs rather than depressed biogenic silica burial flux following the end-Permian extinction. *Glob. Planet. Chang.* **195**, 103330 (2020).
5. Uno, K., Onoue, T., Hamada, K. & Hamami, S. Palaeomagnetism of Middle Triassic red bedded cherts from Southwest Japan: equatorial palaeolatitude of primary magnetization and widespread secondary magnetization. *Geophys. J. Int.* **189**, 1383–1398 (2012).
6. Tomimatsu, Y., Onoue, T. & Nozaki, T. Stratigraphy and radiolarian ages of stratiform manganese deposits from the Chichibu Belt in the Saiki area, eastern Oita Prefecture, Japan (in Japanese with English abstract). *J. Geol. Soc. Japan* **122**, 267–273 (2016).
7. Tomimatsu, Y., Onoue, T. & Rigo, M. Conodont and radiolarian biostratigraphic age constraints on Carnian (Upper Triassic) chert-hosted stratiform manganese deposits from Panthalassa: Formation of deep-sea mineral resources during the Carnian pluvial episode. *Mar. Micropaleontol.* **171**, 102084 (2022).
8. Kambe, N. & Teraoka, Y. Geology of the Usuki district (in Japanese with English abstract). In *Geological Sheet Map at 1:50,000*. 1–78 (Geological Survey of Japan, 1968).
9. Yamashita, D., Kato, H., Onoue, T. & Suzuki, N. Integrated Upper Triassic conodont and radiolarian biostratigraphies of the Panthalassa Ocean. *Paleontol. Res.* **22**, 167–197 (2018).
10. Tomimatsu, Y. et al. Marine osmium isotope record during the Carnian “pluvial episode” (late Triassic) in the pelagic Panthalassa Ocean. *Glob. Planet. Chang.* **197**, 103387 (2021).
11. Ishiga, H. Two suites of stratigraphic succession within the Tamba Group in the western part of the Tamba belt, Southwest Japan (in Japanese with English abstract). *J. Geol. Soc. Japan* **89**, 443–454 (1983).
12. Kimura, K., Nakae, S. & Takahashi, Y. Geology of the Yotsuya distinct (in Japanese with English abstract). In *Geological Sheet Map at 1:50,000*. 1–52 (Geological Survey of Japan, 1994).
13. Nakae, S. Jurassic accretionary complex of the Tamba Terrane, Southwest Japan, and its formative process. *J. Geosci. Osaka City Univ.* **36**, 15–70 (1993).
14. Nakae, S., 2000. Regional correlation of the Jurassic accretionary complex in the Inner Zone of Southwest Japan (in Japanese with English abstract). *Mem. Geol. Soc. Japan* **55**, 73–98 (2000).
15. Wakita, K. Origin of chaotically mixed rock bodies in the early Jurassic to early cretaceous sedimentary complex of the Mino terrane, Central Japan. *Bul. Geol. Surv. Japan* **39**, 675–757 (1988).
16. Wakita, K. Geology of the Tanigumi district (in Japanese with English abstract). In *Geological Sheet Map at 1:50,000*. 1–50 (Geological Survey of Japan, 1991).
17. Yamagata, T. Mesozoic chaotic formations of Mino terrane, Northwestern Mino mountains, Central Japan. *J. Geol. Soc. Japan* **95**, 447–462 (1989).
18. Kojima, S. et al. 2016. In *The Geology of Japa*n (eds Moreno, T., Wallis, S., Kojima, T., Gibbons, W.) 61–100 (Geological Society of London, 2016).
19. Takahashi, I. Geology and manganese ore deposits of the Otaniyama mine, Iwate Prefecture (in Japanese). *J. Japan. Assoc. Mineral. Petrol. Econ. Geol.* **45**, 137–148 (1961).
20. Yoshida, T. & Katada, M. Geology of the Otsuchi district (in Japanese with English abstract). In *Geological Sheet Map at 1:50,000*. 1–30 (Geological Survey of Japan, 1964).
21. Okami, K. Depositional age and environment of bedded manganese deposits distributed in Otaniyama mine area, southeastern part of the northern Kitakami massif, Northeast Japan (in Japanese with English abstract). *Mining Geol.* **40**, 257–268 (1990).
22. Sun, Y.D. et al. Climate warming, euxinia and carbon isotope perturbations during the Carnian (Triassic) Crisis in South China. *Earth Planet. Sci. Lett.* **444**, 88–100 (2016).
23. Zhang, Z.T., Joachimski, M.M., Grasby, S.E. & Sun, Y.D. Intensive ocean anoxia and large δ^13^C_carb_ perturbations during the Carnian Humid Episode (Late Triassic) in Southwest China. *Glob. Planet. Change* **217**, 103942 (2022).
24. Dal Corso, J. et al. Discovery of a major negative δ^13^C spike in the Carnian (Late Triassic) linked to the eruption of Wrangellia flood basalts. *Geology* **40**, 79–82 (2012).
25. Dal Corso, J. et al. Carbon isotope records reveal synchronicity between carbon cycle perturbation and the “Carnian Pluvial Event” in the Tethys realm (Late Triassic). *Glob. Planet. Chang.* **127**, 79–90 (2015).
26. Dal Corso, J. et al. Multiple negative carbon-isotope excursions during the Carnian Pluvial Episode (late Triassic). *Earth-Sci. Rev.* **185**, 732–750 (2018).
27. Hornung, T., Brandner, R., Krystyn, L., Joachimski, M.M. & Keim, L. Multistratigraphic constraints on the NW Tethyan “Carnian Crisis”. *N. M. Mus. Nat. Hist. Sci. Bull.* **4**, 9–67 (2007).
28. Rigo, M. & Joachimski, M.M. Palaeoecology of Late Triassic conodonts: constraints from oxygen isotopes in biogenic apatite. *Acta Palaeontol. Pol.* **55**, 471–478 (2010).
29. Trotter, J.A., Williams, I.S., Nicora, A., Mazza, M. & Rigo, M. Long-term cycles of Triassic climate change: a new δ^18^O record from conodont apatite. *Earth Planet. Sci. Lett.* **415**, 165–174 (2015).
